# Keratosis Obturans—Laminated Epithelial Plug of External Auditory Canal

**Published:** 1887-12

**Authors:** S. Latimer Phillips

**Affiliations:** Savannah, Ga., Formerly House Surgeon to Presbyterian Eye, Ear and Throat Charity Hospital, Baltimore, Md.


### KERATOSIS OBTURANS--LAMINATED EPITHELIAL PLUG OF EXTERNAL AUDITORY CANAL.

BY S. LATIMER PHILLIPS, M. D., SAVANNAH, GA., FORMERLY HOUSE SURGEON TO PRESBYTERIAN EYE, EAR AND THROAT CHARITY HOSPITAL, BALTIMORE, MD.

Thirteen years ago, Wreden, of St. Petersburg, described for the first time a peculiar laminated epithelial plug of the external auditory canal, which he called keratosis obturans to distinguish it from the well-known cerumenosis obturans, or simple impaction of wax in the canal. Keratosis obturans is not common, and when it is met with is apt to be taken for ordinary inspissated cerumen. In the annual report of the Massachusetts Eye and Ear Infirmary for the year 1886, in the 3,261 ear cases treated I find only four cases of this affection. In the Manhattan Eye and Ear Hospital, and the New York Ophthalmic and Aural Institute, I find none, showing its comparative rarity.

It differs greatly from the common impaction of wax, in that it is not dissolved in water as this, and that it is far more difficult to remove. It being impossible to get this plug out of its fixed position with any amount of syringing, other means have to be resorted to. Often the most intense pain is experienced in the canal and round about the ear, neuralgic in character, sometimes extending to the temporal region, and thus misleading the inexperienced. The deafness is gradual in its onset—that is, in the cases I have seen—and is varying in degree.

I will report an illustrative case below which occurred recently in my practice:

Mr. J., merchant, forty-two years of age, in passably fair health, with a tendency to furuncular formations over face and neck, good habits, but of nervous temperament, consulted me, April 11th, 1887, for a painful affection of his left ear, with deafness. He stated that three years since he suffered intensely with pain in left ear and side of head and face. His suffering was so great that it was impossible for him to obtain rest or sleep without an opiate. Several physicians treated him at that time for neuralgia, but he got no relief whatever. His hearing failing him, he consulted an aurist, who removed from his ear a "large plug of white, leathery-looking substance," after many attempts, with marked relief as to pain and hearing. He got on all right up to a year from the time he consulted me, when he had a return of the pain in and about the ear, with hardness of hearing.

On inspection, the canal was found blocked up with a mass of what I took to be wax. There was some yellowish discharge with rather an offensive smell. Hearing reduced to the tick of watch at six inches. I attempted to remove the plug in the usual manner with a syringe and water, but it did not budge. Some small flakes of wax and a few epithelial scales came out with the water. After having thrown in the ear a large number of syringefuls of water, and the mass remaining as firmly fixed as ever, I decided that other means had to be adopted. With a very delicate pair of forceps (the more delicate an ear instrument the better it will serve its purpose), bent at right angles, the canal being well illuminated by the head-mirror, I began by piecemeal to remove the plug. There was intense pain and a great deal of bleeding during the operation. The suffering was so great that he was unable to have the whole removed at one sitting. I gave him a solution of sod. bicarb. (gr. xx. to ℥i aq.), to be warmed and used in the ear freely. This he did, which so softened the mass that after several attempts the whole was removed. The skin in the canal wherever the membrane had been was in an acute stage of inflammation. On the posterior and superior bony portion of the canal, near the membrana tympani, was a small spot of ulceration in the skin. The pressure of the epithelial plug on the skin in this inflamed and sensitive condition accounts for the great pain he suffered. The membrana tympani was somewhat sunken, and had lost much of its normal luster. Hearing improved to watch at eleven inches. In the right ear a yellowish membrane was seen partially filling the canal and extending over the surface of the drum membrane. Watch heard ticking at fourteen inches. This ear was treated in a similar manner to the left, with the exception that I used a 4 per cent. sol. cocaine in removing the membrane (epithelial), after which I had no more pain during my manipulations. Two-thirds of the membrane came away in one piece, the exact shape of the canal and resembling a very small glove-finger. The outer side, or that lying in contact with the skin of the canal, was smooth and white, like dressed kid. It was put in a solution of alcohol, and instead of dissolving like the usual ceruminous plug, holds its shape to-day, six months after its removal, as perfectly as the day it was put there. There was no ulceration found in this canal, but the skin was much inflamed. Drum membrane in fair condition; hearing brought to twenty-four inches.

The inflammation of the skin of the canals was allayed by blowing over the surface a powder composed of boracic acid ℥i., resorcin ʒi., which is very soothing when applied to the parts, and will soon heal them.

No cause as yet has been suggested for this disease. It may be possible that the bad habit of picking in the ear with toothpicks, ear-swabs and such may induce chronic inflammation of the skin of the part, with its consequent desquamation of epithelial scales, these being impacted, one layer upon another, and together with any secretions which take place, forming a mass which fits closely to the parts and exerts an undue pressure upon the inflamed skin with its highly sensitive nerves.

This mass, on being washed from the ear, will have the appearance of wet white tissue paper, and the same boggy feel of wet paper when taken between the fingers and crushed.

The prognosis is usually good, and except in cases where there is pre-existing middle-ear catarrh the hearing will be much improved after the removal of the mass. Patients affected with it are liable to have a return of the trouble, and particularly when they have the habit of picking at their ears. Those with a tendency to this disease should have their ears examined frequently (six months to a year) so as to prevent its going too far before an attempt is made for its removal.

